# Evaluation of exotic soybean accessions and their use in developing improved soybean lines with resistance to Phomopsis seed decay

**DOI:** 10.1371/journal.pone.0286519

**Published:** 2023-06-09

**Authors:** Shuxian Li, James R. Smith, Lingxiao Zhang

**Affiliations:** 1 United States Department of Agriculture, Agricultural Research Service (USDA, ARS), Crop Genetics Research Unit, Stoneville, Mississippi, United States of America; 2 Mississippi State University, Delta Research and Extension Center, Stoneville, Mississippi, United States of America; National Taiwan University, TAIWAN

## Abstract

Poor seed quality of soybean is often associated with Phomopsis seed decay (PSD), which is one of the most economically important seed diseases. *Diaporthe longicolla* (syn. *Phomopsis longicolla*) is the primary cause of PSD. Control of PSD is best accomplished by planting PSD-resistant cultivars. Sixteen exotic soybean accessions from the USDA soybean germplasm collection were screened for reaction to PSD at Stoneville, Mississippi. They consisted of maturity groups (MG) II, III and IV. Seeds from inoculated and non-inoculated plots harvested either promptly at maturity, or after a two-week delay in harvest, were assessed for infection by *D*. *longicolla*. Seed infection ranged from 0 to 36.7%. Overall, PI 417050 (MG II), PI 417017 (MG III), and PI 594692 (MG IV) had significantly (*P* ≤ 0.05) lower percentages of seed infected by *D*. *longicolla* and higher seed germinations than other genotypes in the same maturity groups. PI 587982A also performed well. As a result of these findings, these resistant accessions were used over multiple cycles of breeding to develop improved breeding lines with resistance to PSD and low seed damage. Breeding line 11043-225-72, with combined resistance from both PIs 417050 and 587982A, had low scores for PSD (6.7%) and seed damage (3.4%), while DS65-1, deriving resistance from PI 587982A, had the lowest seed damage score (1.1%) and the highest seed germination (85.6%) among all lines tested in 2017. DS65-1 and 11043-225-72, along with five other improved breeding lines, were provided to public soybean breeders for developing improved cultivars and germplasm lines. DS31-243 (PI 700941), derived from PI 587982A, was publicly released by the USDA in 2022. This research will lead to future releases of improved germplasm lines and cultivars with PSD resistance and high seed quality. It will also aid in disease management and be a benefit to soybean producers and the industry at large.

## Introduction

Soybean [*Glycine max (L*.*)* Merr.] is one of the most economically important legume crops grown world-wide [1]. It provides plant-based protein and oil for animal and human nutrition. Soybean is also valuable for making biodiesel and various industrial products. The capabilities of nitrogen fixation make soybean important in sustainable agricultural practices [2,3]. The global demand for soybean has increased dramatically [4]. However, soybean diseases can cause significant yield losses [http://extension.cropsciences.illinois.edu/fieldcrops/diseases/yield_reductions.php]. Poor seed quality of soybean is often associated with the disease Phomopsis seed decay (PSD), which is one of the most economically important seed diseases of soybean [5–8]. PSD occurs in most soybean production areas worldwide, especially in the mid-southern region of the United States [8–10].

The causal agent of PSD was first identified as *Phomopsis longicolla* in 1985 [5]. However, the fungus was renamed *Diaporthe longicolla* (Hobbs) J. M. Santos (syn. *Phomopsis longicolla*) [11], while the name of the disease was retained. Although *D*. *longicolla* is the primary cause of PSD, different fungi in the *Diaporthe*-*Phomopsis* complex can also cause PSD [7,8]. Several studies have indicated that the isolation frequency of *D*. *longicolla* from soybean has been much higher than that of other fungi associated with PSD. For example, in a three-year study from 2002 to 2004 in Stoneville, Mississippi, *Diaporthe aspalathi* was recovered at very low frequencies, whereas *P*. *longicolla* was the major fungal species with highest isolation frequency [12]. Analysis of a total of 17,280 isolates of three pathogens from the *Diaporthe*-*Phomopsis* complex from diseased plants collected at nine locations each year from 2002 through 2004 in Ontario, Canada indicated that *P*. *longicolla* was the predominant species (41% of isolates), followed by *D*. *phaseolorum* var. *caulivora* (37%) and *D*. *phaseolorum* var. *sojae* (22%) [13]. In a study of *Diaporthe* species associated with symptomatic and asymptomatic infection of soybean stems in Minnesota, *D*. *longicolla* was the most widespread in distribution [14]. Moreover, a single seed could be infected by “either one pathogen or any combination of *D*. *phaseolorum* var. *caulivora*, *D*. *phaseolorum* var. *sojae*, and *P*. *longicolla”* [15]. Thus, PSD can be the result of a complex interaction of fungal species rather than just a single entity. In addition, differences in aggressiveness of *D*. *longicolla* isolates on soybeans from different geographic regions have been observed [16,17]. The genome sequences and protein networks in the pathogen were also analyzed and reported [18–21].

The most significant symptoms of PSD include, but are not limited to, seed discoloration, shriveled and elongated seeds, and cracked seed coats. If soybean seed are severely infected with *D*. *longicolla*, the entire seed surface can be moldy and have a chalk-white color. However, infected soybean seed may also have no visible symptoms [8,22]. It has been reported that symptomless soybean seed with *Phomopsis* infections can still cause poor emergence, reduced seedling vigor, and damping off and plant death, similar to seeds with symptoms [8,23]. Moreover, soybean seed oil quality can be reduced, and other seed components negatively altered, by the presence of *D*. *longicolla* [24].

Environmental conditions play a crucial role in the development of PSD. During the later reproductive growth stages from pod formation through maturation, warm humid environments promote the growth of *D*. *longicolla*, which can then cause severe seed infection [25–27]. While being variable in terms of environments and years, PSD has caused substantial yield losses of soybean [28–30]. In 1994, soybean yield losses of almost two million metric tons (MMT) were caused by PSD in the ten largest soybean producing countries [8]. From 1996 to 2007, soybean yield reductions from PSD were estimated to be about 0.4 MMT [30]. However, in 2010 and 2011 in the midsouthern United States (Arkansas, Mississippi, and Missouri), there was a lack of appreciable soybean infection by *D*. *longicolla* due to the seed maturation process occurring during hot dry weather [10].

Fungicide treatment represents one of the multiple chemical and cultural approaches for the control of PSD [31–34]. In addition, seed infection could be reduced if soybean seed were harvested timely at maturity, as opposed to delaying harvest due to rainy weather. However, utilizing host genetic resistance by planting resistant cultivars is one of the most effective methods for managing PSD in an environmentally friendly way [35–39].

Our long-term research goal is to develop high-yielding soybean cultivars with resistance to soybean diseases. To achieve this goal, the specific objectives of this study were (i) to evaluate unique exotic soybean accessions from the USDA germplasm collection for their reactions to *D*. *longicolla* infection after inoculation, followed by either prompt harvest at maturity or by a two-week delay in harvest after maturity; and (ii) to develop improved soybean breeding lines with PSD resistance and high seed quality.

## Materials and methods

### Germplasm evaluation

Sixteen soybean plant introductions (PI) from the USDA Soybean Germplasm Collection were used in this study (Table 1). Selection of these soybean lines was based on our previous screening of seed quality including, but not limited to, seed germination, frequency of hard seed and wrinkled seed, as well as the incidence of Phomopsis seed decay (PSD) in high-temperature environments with natural infection of PSD [40]. The selected soybean accessions consisted of maturity groups (MG) II, III, and IV, and included the susceptible checks PI 597412 [https://npgsweb.ars-grin.gov/gringlobal/methodaccession?id1=51091&id2=494138] for MG II, PI 417077 for MG III trials [40], and PI 80837 for MG IV [41]. The resistant checks included PI 417274 [42], PI 417017 [43], and PI 417479 [39] for the trials of MG II, III, and IV, respectively.

**Table 1:** A list of exotic soybean accessions from the USDA germplasm collection evaluated for resistance to *Diaporthe longicolla* in this study.

Genotype	MG^w^	Seed coat color	Province	Country
PI 416875	II	Light green	Kyushu	Japan
PI 417050	II	Yellow	Kyushu	Japan
PI 417321	II	Light green	Kyushu	Japan
PI 423941	II	Yellow	Kumamoto	Japan
PI 417274 ^x^	II	Yellow	Kyushu	Japan
PI 597412^y^	II	Yellow	Sichuan	China
PI 85009–1	III	Green	Saitama	Japan
PI 417017 ^x^	III	Yellow	Kyushu	Japan
PI 417328	III	Yellow	Kyushu	Japan
PI 417077 ^y^	III	Yellow	Kyushu	Japan
PI 587982A	III	Yellow	Sichuan	China
PI 594692	IV	Yellow	Guizhou	China
PI 587576	IV	Yellow	Jiangsu	China
PI 594872	IV	Brown	Yunnan	China
PI 80837 ^y^	IV	Yellow	Akita	Japan
PI 417479 ^x^	IV	Yellow	Tohoku	Japan

^w^ Maturity group.

^x^ Resistant check.

^y^ Susceptible check.

Field experiments were conducted at Stoneville, Mississippi (MS) on a Sharkey clay soil (very-fine, smectitic, thermictic Chromic Epiaquert) from 2006 to 2007. For the germplasm evaluation experiments, single-row plots were planted on 18 April 2006 and 20 April 2007. The seeding rate was 33 seeds/m of row in 2.74 m-long rows, with a 0.91-m row spacing. Each plot was a single genotype with a specific inoculation/harvest date treatment.

Maturity groups II, II, and IV were utilized and evaluated as three separate experiments. Within each of these experiments, there was one inoculated block and one non-inoculated block. Each experiment of maturity group and inoculation was a split-plot arrangement of treatments in a randomized complete block design (RCBD), with soybean genotypes as main plots, being randomly arranged within each of six replications, and harvest time (prompt versus two-week delay) as sub-plots.

Each plot was manually bulk-harvested at the appropriate time. Specifically, in 2006, the following treatment combinations were carried out: prompt harvest-inoculated, prompt harvest-non-inoculated, and delayed harvest-inoculated. In 2007, the same treatment combinations were employed with the added combination of delayed harvest-non-inoculated. No chemical treatments were applied to the experiments, as their application to plots inoculated with a soybean pathogen could bias the PSD results. Hence, weed control was manual.

At the R5 growth stage [44], inoculum of *D*. *longicolla*, as described below, was applied twice each year at an interval of 14 days. To promote pathogen infection, water was applied as needed by either manually spraying with a battery-operated Solo Model 416 backpack sprayer (Flojet Co., Santa Ana, CA) or through furrow irrigation.

Weather data of total precipitation, number of rainy days, average maximum temperatures and maximum relative humidity during the soybean growing seasons were obtained from the Stoneville, MS weather station

[http://deltaweather.extension.msstate.edu/ag-weather-tools].

### Breeding populations

As part of an ongoing effort to incorporate the highest levels of resistance from diverse exotic accessions into improved breeding lines, over 100 heterogeneous breeding lines were evaluated annually over multiple years. Crosses were made between high-yielding soybean lines and the newly identified exotic sources of PSD-resistance, as well as between the new sources of resistance to PSD and previously developed heat-tolerant soybeans [45,46]. Although hundreds of breeding lines were developed and tested during the course of the project [47], this work focuses on the results of the following seven improved breeding lines: 11043-225-72, 11043-224-91, 11030-541-28, 10061-236-11, 10076-121-11, DS65-1, and DS31-243. Each of these lines was derived from resistant accessions tested above (PI 587982A and PI 417050) as well as from an additional resistant accession, PI 424324B, that was identified previously by Li et al. [9]. Attempts were also made to create improved lines derived from multiple sources of resistance, such as PI 587982A with PI 424324B and PI 587982A with PI 417050. Susceptible lines were dropped when identified, while the best of these lines went on for additional testing in yield trials and were later shared with public soybean breeders. Lines 11043-225-72 and 11043-224-91 were derived from DS25-1 x PI 417050. DS25-1 [45,46] is an improved heat tolerant soybean germplasm line released by the USDA in 2017 [https://npgsweb.ars-grin.gov/gringlobal/accessiondetail?id=1954416]. It was derived from DT98-9102 x PI 587982A, where PI 587982A was used in the cross because of its heat tolerance, high germinability, and potential resistance to PSD [40]. DT98-9102 is an improved high-yielding germplasm line developed by R. L. Paris at Stoneville, MS and released by USDA, but not entered into the USDA germplasm collection. PI 417050 had high germinability and low PSD in a previous study [40]. Breeding line 11030-541-28 was derived from DS25-1 x PI 424324B, where PI 424324B was previously identified to be resistant to PSD [9]. Line 10061-236-11 was derived from breeding line 04030-1-4-5-1 x LG03-4561-14 [48]. Breeding line 04030-1-4-1-1 [49] is heat tolerant and was derived from cultivar 5601T [50] x PI 587982A. Breeding line 10076-121-11 was derived from line 07055-2-3-7 x ‘Osage’ [51], where breeding line 07055-2-3-7 was derived from one backcross with DT97-4290 [52] x PI 587982A. Breeding line DS65-1 was derived from PI 587982A x S99-11509 [53] and breeding line DS31-243 was derived from LG04-1459 x 07055-2-3-7. LG04-1459 is a high-yielding, exotically-derived unreleased breeding line developed by R. L. Nelson (USDA, retired) at Urbana, IL. All crosses were made at Stoneville, MS during summer seasons and the F_1_ plants were grown in Puerto Rico during the following winter/spring of the year.

Plants were advanced from the F_2_ to F_3_, F_3_ to F_4_, F_4_ to F_5_, F_5_ to F_6_ and any succeeding generations by pedigree selection [54] at Stoneville, MS. In each generation, selection of individual plants was based on the best agronomic traits (lodging, height, lack of shattering, pod load, pod color, etc.) in the field, followed by assays for germination and/or PSD in the lab. Only plant progenies with the best agronomic traits, seed germination, and low PSD scores were advanced to the next generation.

In single-replicated field trials in 2015 and 2016, each plot was a single row 2.7 m long and 0.91 m wide. All plots were bordered with other experimental lines or with ‘Williams 82.’ Twenty-five seed m^-1^ of row were planted at a depth of 2.5 cm using a tractor-drawn planter fitted with seed cone attachments and a hydraulic depth-control system. Plots were furrow irrigated as needed every 10–14 days throughout the growing season, which created humidity that rose from the soil through the plant canopy.

Plants of breeding lines were inoculated twice during the period from R5 through R6 [44]. Single plants were selected from heterogeneous F_3_, F_4_, F_5_ and later-generation rows with the best agronomic traits shortly after R8. Homogeneous rows at the F_5_ and later generations were harvested in bulk. Harvested plant materials were placed in an air-conditioned humidity-controlled building, where they were stored and allowed to uniformly dry, preparatory to being threshed in a single-plant or bundle thresher as appropriate. Once threshed, seed was stored at 21°C and 60% relative humidity until they were assayed for PSD infection and/or seed germination.

In 2017, advanced breeding lines 11043-225-72, 11043-224-91, 11030-541-28, 10061-236-11, 10076-121-11, and DS65-1, and commercial cultivars Pioneer 94Y23, AG4403, AG4232, and Progeny 4211, were grown in replicated four-row plots at Stoneville, MS. Planting date was 10 April and row length was 5.79 m long, but the other planting protocols were as described above. The experimental design was a randomized complete block design with three replications. Due to excessive rains and warm temperatures during August and September in 2017, plots were not inoculated or delay-harvested. The middle two rows of each plot were promptly harvested shortly after R8. Harvested seed was then stored and assayed as per the protocols below. Seed were assayed for PSD, standard germination, and damage kernel total (DKT), as defined by the Federal Grain Inspection Service [https://www.ams.usda.gov/about-ams/programs-offices/federal-grain-inspection-service]. To determine total seed damage (DKT), seed were visually rated for mold, stink bug feeding, green seed, weathering, and heat damage on seed lots of 125 g. Percent seed damage represents the total weight of damaged seed divided by 125 g x 100.

The seven soybean lines (11043-225-72, 11043-224-91, 11030-541-28, 10061-236-11, 10076-121-11, DS65-1, and DS31-243) have been used by colleagues in public breeding programs for developing improved cultivars and germplasm lines. All seven lines have PI 587982A in their background. Lines 11043-225-72 and 11043-224-91 have both PIs 587982A and 417050 in their backgrounds, and line 11030-541-228 has both PIs 587982A and 424324B in its background.

### Inoculum preparation and application

Isolate LiMS-1 of *D*. *longicoll*a, which was available in 2006, was used for inoculation in the germplasm evaluation experiments, whereas another isolate MSPL 10–6 was used for the breeding trials. MSPL 10–6 was one of the most aggressive isolates in our tests [17]. Both isolates were originally from field-grown soybean plants at Stoneville, MS. To prepare the inoculum, the fungal isolate was grown at 24°C on potato dextrose agar (Difco Laboratories, Detroit, MI). After autoclaving, the medium was adjusted to pH 4.8 with 25% lactic acid (APDA). Inoculum was prepared using our previously reported method [9,10]. Briefly, sporulation of the culture was induced under a fluorescent light output of 300 μmol m ^-2^ s^-1^ with a 12-h photoperiod for 30 to 45 days. Sporulating cultures in Petri dishes were then flooded with sterile deionized water three times, agitated to dislodge conidiospores and filtered with four layers of sterile cheesecloth to eliminate the agar. Concentrations of conidiospores were adjusted to approximately 1.5 x 10^5^/ml, which was determined using a hemacytometer (Hausser Scientific, Blue Bell, PA). A battery-operated backpack sprayer (Solo Model 416; Flojet Co., Santa Ana, CA) was used for field inoculations. The sprayer had a hand-held boom containing a single nozzle with an adjustable orifice at 207 kPa. In each plot, a conidiospore suspension was sprayed directly onto the soybean pods and then evenly across the foliage. Plots were sprayed until runoff [9,10,55]. Approximately 500 ml of the conidiospore suspension was applied to each plot.

### Seed assays

Prior to planting, 25 seeds of each line were plated and assayed for incidence of *D*. *longicolla*. After manual harvest and threshing of plants, 25 randomly chosen seeds (13% moisture) from each plot (*i*.*e*. each replication) for each soybean line for each inoculation treatment/harvest time combination were assayed to determine the percent seed infection by *D*. *longicolla*, using the method previously reported [9,10,55]. Seeds were surface-disinfected in 0.5% sodium hypochlorite for 3 min, rinsed in sterile distilled water, and then placed on APDA [9,10,55]. Five seeds were placed on APDA in each 100 mm x 15 mm Petri dish, where one seed was placed in the center and the others were placed equidistantly around the outside of the dish, approximately 10 mm from the side with approximately 30 mm between seeds. All seed plates were incubated for four days at 24°C. The number of seed infected with *D*. *longicolla* was recorded and calculated as percent seed infection. Seed germination of 200 arbitrarily selected seeds from each line was determined using a standard soybean seed germination protocol [56].

### Isolate identification

Putative *D*. *longicolla* colonies were transferred to new APDA plates. In order to distinguish *D*. *longicolla* from other *Diaporthe* spp. in the *Diaporthe*-*Phomopsis* complex, such as *D*. *sojae* (syn. *Phomopsis phaseoli*), *Diaporthe caulivora* and *Diaporthe aspalathi* (syn. *Diaporthe phaseolorum var*. *meridionalis*), thirty selected seed plates with putative *D*. *longicolla* colonies were maintained for 45 days at 24°C under 12-h light. In addition, 10 putative *D*. *longicolla* isolates, along with the type strain TWH P74, LiMS-1, and isolate MS-SSC91 of *D*. *aspalathi* (syn. *Diaporthe phaseolorum var*. *meridionalis*) [57] were transferred to PDA or water agar with autoclaved soybean stem pieces or Williams 82 seeds for 45 days under incubation conditions. Observations under the Olympus SZX12 dissecting microscope were conducted beginning 10 days after incubation to check if any isolates formed perithecia. Perithecia have not been found in *D*. *longicolla*, but have been reported to be produced by other fungal pathogens in the *Diaporthe-Phomopsis* complex, such as *D*. *aspalathi* that causes southern soybean stem canker, and *D*. *caulivora* (syn. *Diaporthe phaseolorum var*. *caulivora*) that causes northern soybean stem canker [58], as well as *D*. *sojae* (syn. *Phomopsis phaseoli*) that causes soybean pod and stem blight [59].

As we previously reported [55], identification of purified isolates of *D*. *longicolla* was conducted using both cultural morphology and molecular approaches. Initially, morphological characteristics including conidial type, sporulation, and stromal pattern were performed according to Hobbs et al. [5]. Analysis of the rDNA sequences of the internal transcribed space (ITS) regions and the translation elongation factor 1-α gene (TEF-1 α) were performed to confirm the identity of the isolates of *D*. *longicolla*. Two primers of ITS1 (5’-TCCGTAGGTGAACCTGCGG-3’) and ITS4 (5’-TCCTCCGCTTATTGATATGC-3’) were used to amplify the ITS region by PCR [55,60,61], while primers of EF1-728F (5’- CAT CGA GAA GTT CGA GAA GG -3’) and EF1-986R (5’-TAC TTG AAG GAA CCC TTA CC -3’) were used to amplify the TEF-1 α gene [60]. All sequences were analyzed using BLASTn [55].

### Data analyses

Quantitative data were analyzed statistically and analyses of variance were calculated using the Generalized Linear Mixed procedure (PROC GLMMIX) in SAS (version 9.4, SAS Institute, Cary, NC) with a negative binomial distribution and a log link function specified for seed infection by *D*. *longicolla* [62]. Genotype, inoculation treatment, harvest date, and year were independent variables, whereas incidence of *D*. *longicolla*, percent seed germination, and percent damaged seed (DKT) were dependent variables. Mean percent seed germination was calculated for each entry. Data were combined across years to test if there were interactions between genotype and year when “year” was the fixed effect, and to compare genotypes within a year, in which “year” was the random effect and “genotype” was the fixed effect. The genotypes were compared with Fisher’s least significant difference (LSD) at *P* ≤ 0.05 to determine differences among them. Pearson’s correlation coefficients between percent seed infected by *D*. *longicolla* and percentage of seed germination were computed using the PROC CORR procedure of SAS.

## Results

### Evaluation of exotic accessions for incidence of *D*. *longicolla* and percent seed germination

**Fig 1:**
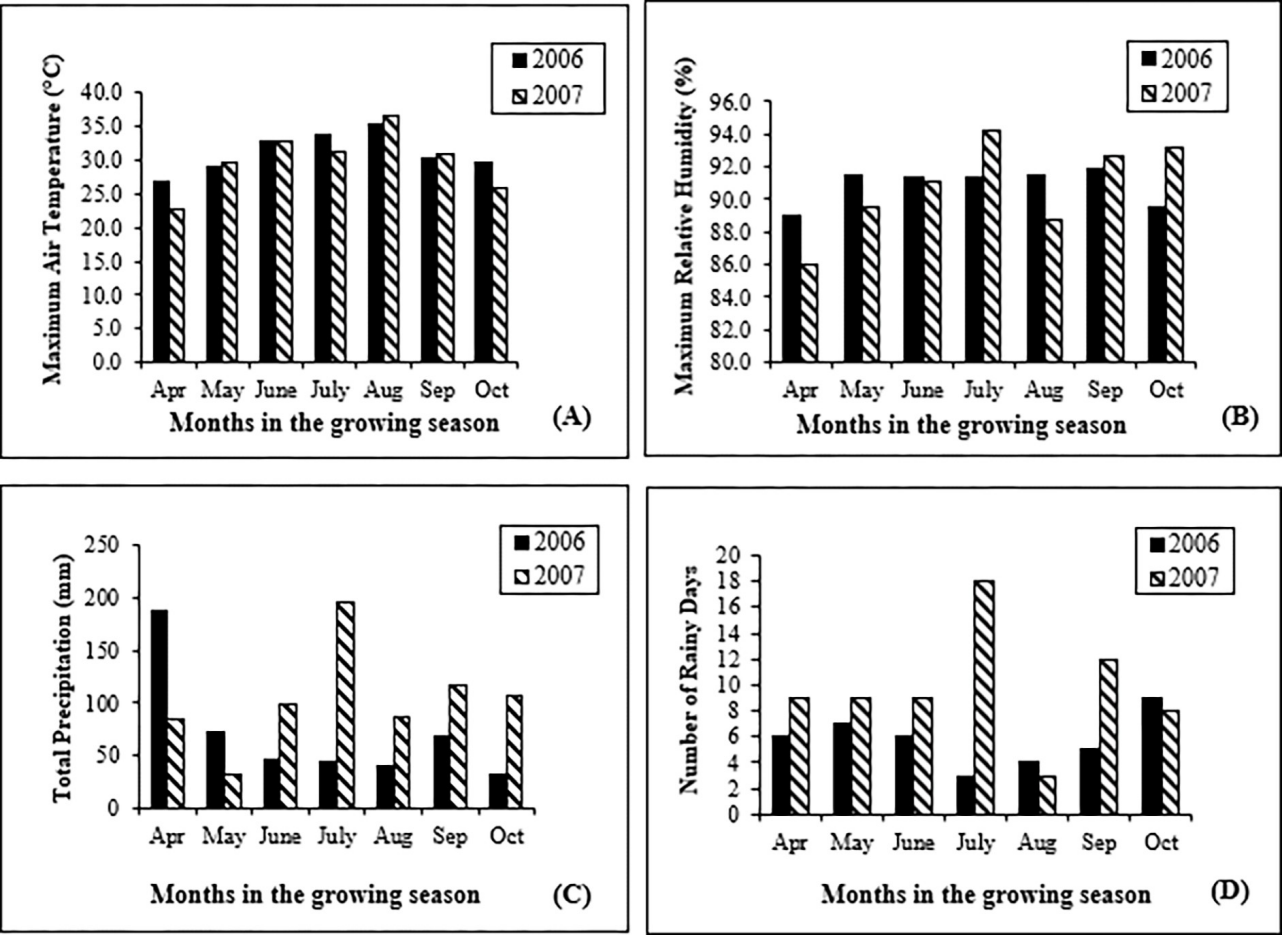
Weather information from Stoneville, MS during soybean growing seasons for the months of April through October in 2006 and 2007. **A:** Air maximum temperatures. **B:** Maximum relative humidity. **C:** Total precipitation. **D:** Number of rainy days.

The weather information during the soybean growing seasons of 2006 and 2007 is shown in Fig 1. The maximum air temperatures for the growing season of April through October in 2006 averaged 31.1°C and ranged from 26.9 to 35.5°C, while in 2007, they averaged 31.9°C with a range from 22.9 to 36.7°C. In August, most of the plants were at the R5 or R6 growth stage. The average daily maximum air temperatures for August in 2006 and 2007 were 35.5°C and 36.7°C, respectively, which were much higher than 24°C, the optimal temperature for PSD. In 2006, totals for precipitation in June, July, and August were 46, 45, and 40 mm, respectively, while in 2007, totals for precipitation in those months were 99, 197, and 87 mm, respectively (Fig 1).

Results of seed assays prior to planting indicated that the soybean accessions we selected had good seed quality. They were free of *D*. *longicolla*. However, the PSD-causing pathogen was found in seeds harvested from the field inoculation experiments. Putative isolates of *D*. *longicolla* that had typical morphology similar to those of the type strain TWH P74 (ATCC 60325) and isolate LiMS-1 were recorded for each seed tested and used to determine the percentage of seed infection by *D*. *longicolla*. Observations of 45-day-old cultures of 30 putative isolates of *D*. *longicolla* under the Olympus SZX12 dissecting microscope, on either PDA plates from the seed plating assay or water agar with soybean stem pieces, indicated that no perithecia were present. However, the stem canker-causing pathogen *D*. *aspalathi*, isolate MS-SSC91, did form perithecia in the side-by-side comparison with isolates of *D*. *longicolla*. Other unidentified *Diaporthe/Phomopsis* spp., as reported by Mengistu et al. [12], as well as *Fusarium* spp. and *Alternaria* spp., were found infrequently. Because soybean plants were inoculated with *D*. *longicolla* in our field experiments, and *D*. *longicolla* is the prevalent species, the data that we present from this study are focused only on this species.

DNA sequences of four selected isolates were identical with our previous *D*. *longicolla* isolates, deposited to Genbank, at the ITS region (Accession MF134860) and in the *TEF-1α* gene (Accession MF189565) [55]. They also have 99–100% identity with many other reported sequences of *D*. *longicolla* strains in GenBank, which included, but were not limited to, accession NR_144924 for the type strain TWH P74 at the ITS regions, and Accessions 590766 and HQ445912 for isolates FAU657 and ER 1678 in the *TEF-1α* gene, respectively. It is notable that when the sequences of our *D*. *longicolla* isolates were compared with *D*. *sojae* isolates, such as FAU499 (Accession KJ590760) and FAU604 (Accession KJ590759), there was only 84% similarity of sequence between those two fungal pathogens.

Analysis of variance of seed infection by *D*. *longicolla* for MG II, MG III and MG IV accessions indicated that there were significant differences (*P* ≤ 0.05) among accessions, harvest times, and years. There were significant (*P* ≤ 0.05) interactions of accession × year, and accession × harvest × year. In MG III and MG IV, interactions of treatment × year were found. For seed germination, there were significant differences (*P* ≤ 0.05) among accessions, harvest time, and years, and the interactions of accession × year, accession × harvest, and accession × harvest × year were significant at *P* ≤ 0.05 (ANOVA table not shown).

Mean percentage infected seeds for maturity groups (MGs) II, III and IV entries are shown in Table 2. The susceptible checks PI 597412 (MG II), PI 417077 (MG III), and PI 80837 (MG IV) had the highest percentage of seed infection by *D*. *longicolla* for almost all inoculation treatments, harvest times, and years, except PI 417479 (MG IV) in the inoculated delayed harvest treatment in 2007. Interestingly, PI 417479 was identified as a resistant source to PSD in an extensive screening project at Columbia, MO, and Isabela, PR [39,63] and was used in developing resistant germplasm line SS93-6181 [38]. In most cases, plants that were inoculated with *D*. *longicolla* had a higher percentage of seed infection by *D*. *longicolla* than non-inoculated plants. For example, in the prompt-harvest non-inoculated treatments in 2006, the percentages of seed infection by *D*. *longicolla* of PI 417321 (MG II), PI 417328 (MG III), and PI 80837 (MG IV) were 0, 0, and 3.3%, respectively, while in the prompt-harvest inoculated treatments, their corresponding percentages of seed infection by *D*. *longicolla* were 5.8, 16.7, and 36.7%, respectively (Table 2). However, PI 417328 had similar percentages of seed infection by *D*. *longicolla* in both the delay-harvest non-inoculated and delay-harvest inoculated treatments, with 12.0% and 12.7%, respectively in 2007 (Table 2).

**Table 2:** Mean percent seed infection by *Diaporthe longicolla* on 16 exotic soybean accessions from maturity groups II, III, and IV in replicated field tests with inoculated and non-inoculated treatments and prompt and delayed-harvest times at Stoneville, Mississippi in 2006 and 2007.

		Phomopsis (%) ^p^															
		2006						2007									
		Prompt ^q^				Delayed ^r^		Prompt				Delayed				Mean^s^	
Genotype	MG^t^	Non^u^		Inoc ^v^		Inoc		Non		Inoc		Non		Inoc			
PI 416875	II	3.3	b ^w^	10.8	ab	9.3	b	3.3	B	2.7	d	6.7	c	4.7	bc	5.8	c
PI 417050	II	0.0	bc	0.8	d	3.3	d	0.0	C	2.7	d	1.6	d	2.0	c	1.5	e
PI 417321	II	0.0	bc	5.8	bc	5.8	bc	0.7	C	1.3	d	2.7	d	3.3	c	2.8	cde
PI 423941	II	0.8	b	0.8	d	5.0	bc	0.0	C	8.0	c	5.6	c	12.8	b	4.7	cd
PI 417274 ^x^	II	0.0	bc	2.5	c	4.2	c	13.3	A	15.3	b	10.7	b	13.6	b	8.5	b
PI 597412 ^y^	II	10.8	a	11.7	a	22.5	a	17.3	A	33.3	a	15.3	a	28.0	a	19.8	a
Mean		2.5		5.4		8.4		5.8		10.6		7.1		10.7		7.2	
PI 85009–1	III	0.8	b	5.8	b	6.7	b	6.0	B	3.3	b	9.3	b	13.3	b	6.5	bc
PI 417017 ^x^	III	0.8	b	5.8	b	5.8	b	1.3	bc	3.3	b	8.0	b	14.0	b	5.6	c
PI 417328	III	0.0	b	16.7	ab	13.3	b	2.7	B	8.0	b	12.0	a	12.7	b	9.3	b
PI 417077 ^y^	III	14.2	a	20.0	a	26.7	a	36.0	A	21.3	a	18.0	a	24.0	a	22.9	a
PI 587982A	III	1.7	b	4.2	b	8.3	b	2.7	B	8.0	b	8.7	b	20.7	a	7.7	bc
Mean		3.5		10.5		12.2		9.7		8.8		11.2		16.9		10.4	
PI 594692	IV	0.8	a	2.5	d	2.5	c	5.3	C	14.7	c	8.0	c	12.0	d	6.5	d
PI 587576	IV	0.8	a	13.3	bc	13.3	b	5.3	C	12.7	c	11.3	c	17.3	c	10.6	c
PI 594872	IV	2.5	a	8.3	c	16.7	b	12.7	ab	8.7	ab	18.0	b	20.0	bc	12.4	b
PI 80837 ^y^	IV	3.3	a	36.7	a	31.7	a	18.0	A	30.7	a	28.7	a	24.7	ab	24.8	a
PI 417479 ^x^	IV	3.3	a	19.2	b	15.0	b	11.3	bc	22.7	bc	19.3	b	29.3	a	17.2	b
Mean	IV	2.2		16.0		15.8		10.5		17.9		17.1		20.7		14.3	

^**p**^ Mean percentage of seed infected by *Diaporthe longicolla* from the seed plating assays and analyzed with a negative binomial distribution and a log link function. Non-inoculated and inoculated data were analyzed separately for each harvest time.

^**q**^ Prompt harvest shortly after soybeans mature at the R8 growth stage.

^**r**^ Delayed harvested at R8 plus 2 weeks. The non-inoculated/delay-harvest treatment was not conducted in 2006.

^s^ Overall mean of non-inoculated and inoculated treatments and harvest times across both 2006 and 2007.

^t^ Maturity group.

^u^ Non-inoculated treatment.

^v^ Inoculated treatment.

^w^ Numbers followed by the same letter within a column in the same maturity group tests are not significantly different by the least significant difference test at *P ≤* 0.05.

^x^ Resistant check.

^y^ Susceptible check.

There was a significant (*P* ≤ 0.05) time-of-harvest effect. In general, *D*. *longicolla* seed infection was higher in plots with delayed harvest than in those with prompt harvest. For example, a susceptible accession PI 597412 (MG II) had 11.7% seed infection when harvested on time and 22.5% infection when delay harvested from the inoculated plots in 2006. Percentages of *D*. *longicolla* seed infection of PI 417017 (MG III) of inoculated plots in 2007 were 3.3% and 14.0% in prompt and delayed-harvest tests, respectively. In the non-inoculated plots, PI 587576 (MG IV) had seed infection of 5.3% and 11.3% in prompt and delayed-harvest tests, respectively (Table 2). However, there were exceptions. For example, PI 594692 had the same 2.5% of seed infection by *D*. *longicolla* in both harvest schemes in non-inoculated plots in 2006.

In view of the results of inoculation treatments and harvest times across both years, PI 417050 (MG II), PI 417017 (MG III), and PI 594692 (MG IV) had significantly (*P* ≤ 0.05) lower percent seed infected by *D*. *longicolla* than most other soybean genotypes in the same maturity group test (Table 2). In addition, soybean genotypes PI 416875, PI 417321, PI 423941 and PI 417274 in MG II, PI 85009–1, PI 417328, and PI 587982A in MG III, and PI 587576, PI 594872 and PI 417479 in MG IV had significantly (*P* ≤ 0.05) lower percent seed infected by *D*. *longicolla* than their respective susceptible checks in their respective maturity group tests (Table 2).

There were differences in seed germination among accessions (Table 3). In general, inoculation with *D*. *longicolla* decreased seed germination. In 2006, percent seed germination of PI 417274 (MG II) in the non-inoculated treatment with prompt harvest was 98.3%, while it was 86.7% in the inoculated test (Table 3). In 2007, with prompt harvest, PI 417017 (MG III) and PI 594692 (MG IV) were other good examples. They had 96.0 and 85.7% seed gemination in the non-inoculated treatment, respectively. However, their percentages of seed germination in the inoculated treatment were both reduced to 77.3% and 57.7%, respectively. In some cases, inoculation treatments did not cause significant differences, such as for the susceptible check PI 417077 in the MG III prompt-harvest trial in 2006, where the percentages of seed germination in both non-inoculated and inoculated tests were 45% (Table 3). Harvest times could also affect seed germination. It appeared that delayed harvest decreased seed germination in most cases. In MG II trials in 2007, PI 417050 had 86.0% and 83.0% seed germination in non-inoculated and inoculated treatments, respectively, when harvested promptly, but 78.4% (non-inoculated) and 76.0% (inoculated) seed germination in the delayed harvests (Table 3).

**Table 3:** Means of seed germination of exotic soybean accessions in replicated field tests at Stoneville, Mississippi in 2006 and 2007.

			Germination (%) ^p^														
		2006						2007								Mean ^q^	
		Prompt ^r^				Delayed ^s^		Prompt					Delayed				
Genotype	MG ^t^	Non ^u^		Inoc ^v^		Inoc		Non		Inoc		Non		Inoc			
PI 416875	II	93.3	b ^**w**^	88.3	ab	84.2	bc	72.3	A	83.3	a	34.7	c	59.3	b	73.6	bc
PI 417050	II	94.2	ab	85.8	b	94.2	a	86.0	A	83.3	a	78.4	a	76.0	a	85.4	a
PI 417321	II	96.7	ab	95.0	a	89.2	ab	76.0	A	84.0	a	62.7	b	67.3	ab	81.6	a
PI 423941	II	95.8	ab	90.8	ab	94.2	a	84.0	A	72.7	a	60.8	b	44.4	c	77.5	ab
PI 417274 ^**x**^	II	98.3	a	86.7	b	77.5	c	54.7	B	62.0	a	49.7	c	58.0	b	69.6	c
PI 597412 ^**y**^	II	61.7	c	41.7	c	16.7	d	41.3	B	30.0	b	32.0	c	33.3	c	36.7	d
Mean		90.0		81.4		76.0		69.1		69.2		53.1		59.0		71.1	
PI 85009–1	III	94.2	ab	85.0	ab	79.2	a	72.0	B	73.3	b	34.7	bc	23.3	b	66.0	b
PI 417017 ^**x**^	III	98.3	a	90.0	a	83.3	a	96.0	A	77.3	b	53.8	a	62.0	a	80.1	a
PI 417328	III	97.5	a	76.7	b	85.8	a	76.0	B	62.0	b	28.8	c	58.7	a	69.4	b
PI 417077 ^**y**^	III	45.0	c	45.0	c	30.0	b	33.3	C	38.0	a	23.3	c	32.7	b	35.3	c
PI 587982A	III	88.3	b	85.0	ab	31.7	b	90.7	A	75.3	b	49.3	ab	67.3	a	69.7	b
Mean		84.7		76.3		62.0		73.6		65.2		38.0		48.8		64.1	
PI 594692	IV	94.2	a	90.0	a	83.3	a	85.7	A	57.7	a	51.2	ab	60.7	a	74.7	a
PI 587576	IV	85.0	b	19.2	b	68.3	b	70.3	A	64.5	a	56.8	a	58.7	a	60.4	b
PI 594872	IV	59.2	d	62.5	d	38.2	c	45.7	C	40.0	bc	43.8	b	28.3	c	45.4	d
PI 80837 ^**z**^	IV	71.7	c	63.3	c	26.3	d	36.0	C	29.8	c	30.2	c	25.3	c	40.4	d
PI 417479 ^**x**^	IV	86.7	ab	33.3	b	65.0	b	64.2	B	51.7	ab	47.5	b	41.7	b	55.7	c
Mean	IV	79.3		53.7		56.2		60.4		48.7		45.9		42.9		55.3	

^**p**^ Percentage of seed germination based on tests of 200 seeds from each soybean accession.

^**q**^ Overall mean of non-inoculated and inoculated treatments and harvest times across both 2006 and 2007.

^**r**^ Promptly harvested shortly after soybeans matured at the R8 growth stage.

^**s**^ Delay harvested two weeks after the R8 growth stage.

^t^ Maturity group.

^u^ Non-inoculated control sprayed with distilled water.

^v^ Plants were inoculated with a spore suspension of *Diaporthe longicolla* (2 x 10^5^) at the R5 growth stage.

^w^ Numbers followed by the same letter within a column in the same maturity test are not significantly different by the least significant difference test at *P ≤* 0.05.

^x^ Resistant check.

^y^ Susceptible check.

^z^ Ancestor check with a history of some susceptibility.

**Fig 2:**
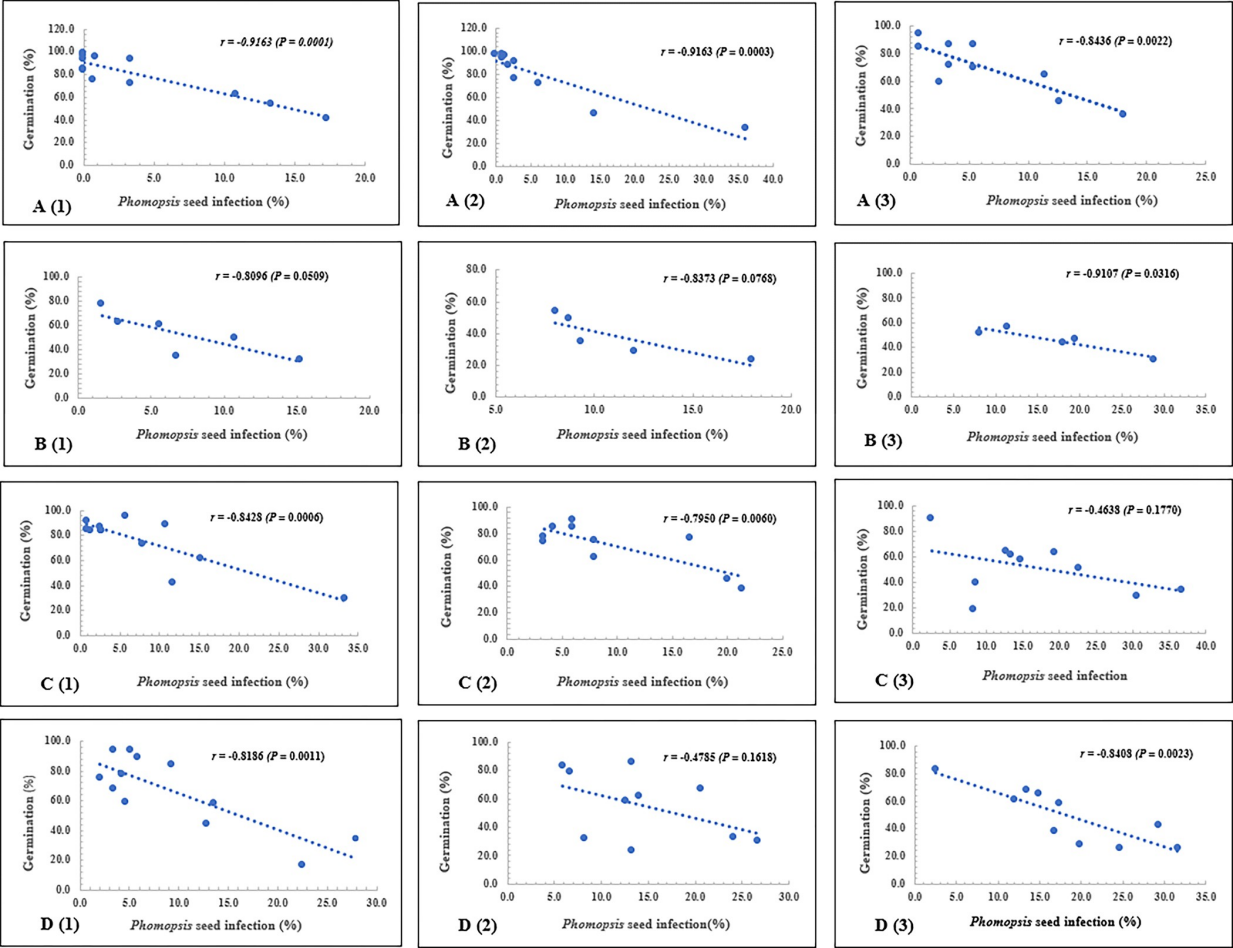
Pearson correlation coefficients between percentage of seed infection by *Diaporthe longicolla* and seed germination in replicated field tests at Stoneville, Mississippi in 2006 and 2007. A. non-inoculation and prompt harvest; B. non-inoculation and delayed harvest; C. inoculation and prompt harvest; D. inoculation and delayed harvest. (1), (2), and (3) are soybean maturity groups II, III and IV, respectively.

Results from correlation analyses indicated that percent seed infected by *D*. *longicolla* was significantly (*P* < 0.05) and negatively correlated with seed germination in almost every inoculation treatment and harvest time across both 2006 and 2007 (Fig 2).

### Evaluation of improved breeding lines

Lines 11043-225-72, 11043-224-91, and 11030-541-28 were tested in 2015 and 2016 for PSD and seed germination. Although the scores were all zero for PSD in 2015 and were low, ranging from 8.0 to 28.0%, in 2016, there were not adequate checks for comparison (Table 4). However, for seed germination, there were multiple appropriate susceptible checks (CZ3841LL, LG03-4561-14, and LD06-7620), with germination scores of 51.5%, 46.0%, and 34.0%, respectively in 2016, and similar scores in 2015, although lacking data for CZ3841LL for that year (Table 4). In comparison, the seven improved breeding lines all had germination percentages in the 90s in both 2015 and 2016, indicating that they all had high germinability and good seed quality in stressful environments.

**Table 4:** Mean percentages of seed infection by *Diaporthe longicolla*, seed germination, and total seed damage (DKT) for seven improved soybean breeding lines derived from exotic accessions and six soybean checks ^u^.

Genotype	PSD (%) ^v^			Germination (%) ^w^			DKT (%) ^x^
	2015	2016	2017^a^	2015	2016	2017	2017
**Breeding lines**							
11043-225-72	0	28.0	6.7	98.0	96.0	75.0	3.4
11043-224-91	0	8.0	62.7	96.0	92.0	46.3	2.7
11030-541-28	0	14.4	44.0	98.0	93.0	73.3	4.8
10061-236-11	NA ^y^	NA	34.7	95.0	93.0	84.7	3.6
10076-121-11	NA	NA	21.3	93.0	92.0	57.7	4.6
DS65-1	NA	NA	37.3	98.0	96.0	85.6	1.1
DS31-243	NA	NA	NA	90.0	94.0	NT ^**z**^	NT
**Released checks**							
Pioneer 94Y23	NT	NT	70.7	NT	NT	15.3	9.9
CZ3841 LL (Late MG III)	NT	NT	NT	NT	51.5	NT	NT
LG03-4561-14 (Late MG III)	NT	NT	NT	47.0	46.0	NT	NT
LD06-7620 (Early IV)	NT	NT	NT	31.0	34.0	NT	NT
AG4403	NT	NT	69.3	NT	NT	29.3	8.5
AG4232	NT	NT	62.7	NT	NT	23.3	15.6
Progeny 4211	NT	NT	54.7	NT	NT	33.3	15.7

^u^ The resistant lines were developed from this study and have been used by public breeding programs for the development of improved cultivars and germplasm lines.

^v^ Percentage of seed infected by *Diaporthe longicolla* from the seed plating assays and analyzed with a negative binomial distribution and a log link function.

^w^ Percentage of seed germination based on tests of 200 seed from each soybean line.

^x “^Damage Kernel Total” was determined by Federal Grain Inspection Services (FGIS) standards for the levels of seed damage (https://www.ams.usda.gov/about-ams/programs-offices/federal-grain-inspection-service).

^y^ Seed were not available because the line had not yet been developed.

^z^ Not tested due to limited seed.

In 2017, replicated plots gave the best seed quality estimates for 11043-225-72, 11043-224-91, 11030-541-28, 10061-236-11, 10076-121-11, and DS65-1. There were no data for DS31-243 in 2017. Breeding line 11043-225-72 had minimal PSD (6.7%) and DKT (3.4%) scores compared to those of commercial cultivars (Pioneer 94Y23, AG4403, AG4232, and Progeny 4211), whose scores ranged from 70.9% to 54.7% for PSD and from 15.7% to 8.5% for DKT. DS65-1 had the lowest DKT score (1.1%), but an intermediate (37.3%) PSD score (Tables 4 and 5). DKT scores of less than 2.1% generally do not receive price discounting at grain elevators due to damage. The four other breeding lines had PSD values that ranged from 21.3 to 62.7%, and DKT values that ranged from 2.7 to 4.8% (Table 4). The highest germination percentage was 85.6% for DS65-1, whereas the scores of the other five breeding lines ranged from 46.3 to 84.7%. Pioneer 94Y23, AG4403, AG4232, and Progeny 4211 had germination percentages of 15.3, 29.3, 23.3, and 33.3%, respectively, indicating a lower level of seed quality and germinability for these cultivars (Table 4). DS31-243 was released by USDA-ARS in 2022 and assigned accession number PI 700941 in the Germplasm Resources Information Network (GRIN). We expect that derivatives from one or more of the other breeding lines will be released in the future.

**Table 5:** Mean percentages of seed germination of putative Phomopsis-resistant soybean breeding lines from field trials in 2015, 2016, and 2017 ^u^.

Breeding line^v^	Pedigree	Generation ^w^	Germination (%) ^x^		
			2015	2016	2017
11030-2-2-4-2-2-1	DS25-1 x PI424324B ^y^	F_6_	96.0	96.0	35.0
11030-2-2-4-2-2-2	DS25-1 x PI424324B	F_6_	96.0	98.0	87.0
11030-2-2-4-2-2-3	DS25-1 x PI424324B	F_6_	96.0	96.0	82.0
11030-2-2-4-2-2-4	DS25-1 x PI424324B	F_6_	96.0	96.0	87.0
11030-2-2-4-2-5-1	DS25-1 x PI424324B	F_6_	92.0	96.0	91.0
11030-2-2-4-4-1-1	DS25-1 x PI424324B	F_6_	96.0	84.0	NT ^z^
11030-2-2-4-4-1-2	DS25-1 x PI424324B	F_6_	96.0	94.0	96.0
11030-2-2-4-4-4-1	DS25-1 x PI424324B	F_6_	96.0	96.0	86.0
11030-2-2-4-4-5-1	DS25-1 x PI424324B	F_6_	98.0	94.0	90.0
11030-2-2-4-4-5-2	DS25-1 x PI424324B	F_6_	98.0	98.0	81.0
11030-4-4-2-2-1-1	DS25-1 x PI424324B	F_6_	96.0	94.0	82.0
11030-4-4-2-6-5-1	DS25-1 x PI424324B	F_6_	94.0	88.0	NT
11030-4-4-2-7-2-1	DS25-1 x PI424324B	F_6_	96.0	86.0	NT
11030-5-2-8-3-3-3-6	DS25-1 x PI424324B	F_7_	96.0	98.0	90.0
11030-5-2-8-5-1-1-1	DS25-1 x PI424324B	F_7_	98.0	88.0	90.0
11030-5-2-8-5-1-1-2	DS25-1 x PI424324B	F_7_	98.0	96.0	88.0
11030-5-2-8-5-1-1-3	DS25-1 x PI424324B	F_7_	98.0	96.0	88.0
11030-5-2-8-5-1-1-4	DS25-1 x PI424324B	F_7_	98.0	96.0	96.0
11030-5-2-8-5-1-1-5	DS25-1 x PI424324B	F_7_	98.0	96.0	90.0
11043-1-1-3-6-5-2	DS25-1 x PI417050	F_6_	96.0	96.0	23.0
11043-225-72	DS25-1 x PI417050	F_5_	98.0	96.0	75.0
11043-224-91	DS25-1 x PI417050	F_5_	96.0	92.0	46.3
11030-541-24	DS25-1 x PI424324B	F_5_	94.0	96.0	74.3
11043-224-81	DS25-1 x PI417050	F_5_	94.0	93.0	64.7
11030-541-28	DS25-1 x PI424324B	F_5_	98.0	93.0	73.3
11030-541-26	DS25-1 x PI424324B	F_5_	96.0	93.0	57.3
11030-541-29	DS25-1 x PI424324B	F_5_	94.0	89.0	62.7
11030-541-210	DS25-1 x PI424324B	F_5_	96.0	94.0	84.3
Mean ± S.E.			96.2 ± 0.4	93.9 ± 1.0	76.8± 5.7

^**u**^ These lines were completely free of Phomopsis seed decay based on seed plating assays in 2014, 2015, and 2016, using F_5_, F_6_, and F_7_ seed, respectively.

^**v**^ Specific soybean breeding line designation is based on pedigree selection. Each successive number after the first hyphen (moving left to right) indicates the plant number selected in the F_1_, F_2_, F_3_, F_4_, F_5_, F_6_, and F_7_, respectively. 11043-2-2-4-8-1 has no F_6_ plant number because the row was bulked as an F_5_-derived row. The sequence of numbers for each line can be used to indicate relatedness among lines. For example, the first four lines were derived from the same F_1_, F_2_, F_3_, F_4_, and F_5_ plant, but different F_6_ plants. These four lines are highly related.

^**w**^ Generation that the lines were bulked.

^**x**^ Germination assays were conducted by the Mississippi Bureau of Plant Industry State Seed Lab per the official protocol for standard germination tests. Due to seed availability, fifty seeds were assayed per line for single plant-derived lines and 200 seeds were assayed for bulked lines.

^**y**^ Phomopsis resistance source, either PI 424324B, PI 417050, or PI587982A. DS25-1 is derived from PI587982A.

^**z**^ Not tested. It was dropped from the program due to poor seed quality.

## Discussion

Seed quality is very important for soybean growers and processors. Poor seed quality of soybean is often associated with Phomopsis seed decay (PSD). In this study, experiments were designed to evaluate 16 maturity group II, III and IV exotic soybean accessions as a first step toward developing improved soybean germplasm lines. The soybean accessions selected for this study showed different levels of natural field infection from *D*. *longicolla* in high-temperature environments in 2002 and 2003 [40]. We therefore hypothesized that PSD-resistant gene(s) may exist in these selected soybean lines. Furthermore, we surmised the new sources of resistance to PSD could be identified under controlled inoculation treatments and utilized for breeding improved germplasm lines with resistance to PSD.

Field trials without inoculation may risk plants escaping pathogen infection if the pathogen is not evenly distributed in the field, which would lead to false conclusions in identifying resistant sources [10,55]. Artificial inoculation under controlled conditions can provide a more uniform distribution of the pathogen and reduce the incidence of disease “escapes” when evaluating a genotype’s reaction to the pathogen. It also provides disease pressure for confirming disease resistance of soybean genotypes [9,64,65]. In this study, soybean genotypes were evaluated under inoculated and non-inoculated treatments. Significant differences in percentages of seed infection by *D*. *longicolla* enabled identification of resistant genotypes to PSD in all three maturity groups of soybeans. PI 417050 (MG II), PI 417017 (MG III), and PI 594692 (MG IV) had significantly (*P* ≤ 0.05) lower percentages of seed infected by *D*. *longicolla* than their respective susceptible checks, as well as compared to other genotypes in the same maturity group tests. There were no specific effects of seed infection, germination, or damage due to maturity group in this study, as maturity and genotype were confounded (each genotype has only one maturity).

Populations with resistant accessions were made in 2006 and tested in subsequent generations. Although not as numerically low in PSD as the above three genotypes, other accessions, such as PI 587982A, also had significantly lower PSD values than the susceptible checks. PI 587982A was used extensively in breeding for PSD and heat resistance and is therefore in the parentage of each of the lines in Table 4. Additionally, PI 417050, in combination with PI 587982A, was used to develop breeding line 11043-225-72, which had among the lowest observed levels of both PSD (6.7%) and DKT (3.4%) among all lines tested in 2017 (Table 4). Further, DS65-1 had the highest germination (85.6%) and the lowest level of DKT (1.1%) in 2017, and was the only line tested that had seed damage levels less than the typical price-discount level of 2.1%. These sources of resistance to PSD are highly useful in soybean breeding programs and have been extensively utilized.

Colonization of *D*. *longicolla* on soybean and development of PSD are very sensitive to environmental conditions and prefer warm and humid conditions [23,26,66]. In this study, the incidence of PSD was higher in 2007 than in 2006, likely because of environmental factors. When examining weather data, average maximum air temperatures during the soybean growing seasons of 2006 and 2007 were similar, but there were large differences in total precipitation. In July, when most soybean plants reached the R5 growth stage and seeds begin to develop in pod cavities [44], the total precipitation in 2006 was 45 mm, while in 2007, it was 196 mm. During the harvest season in August, the total precipitation in 2006 was 45 mm, while in 2007, it was 87 mm. The difference in late season rainfall between 2006 and 2007 was likely the main cause for differences in PSD incidence between those two years.

The "early soybean production system” (ESPS) is very popular in the midsouthern United States [67]. In this system, early maturing cultivars are planted in late-March through April, and mature in August through September. The ESPS allows soybean to take advantage of plentiful spring and early summer rains to increase chances of avoiding late-season drought, thereby increasing soybean yield [67]. However, the weather in August is usually hot and humid, which can result in severe PSD and resulting poor seed germination in the south [68]. Rainfall or moisture after maturity has been reported to be the key factor determining the extent of damage by *D*. *longicolla* and other seedborne pathogens [69]. Delayed harvest due to rainfall with high humidity usually leads to higher infection levels [70]. In this study, the effect of harvest timeliness (prompt vs. delayed) on the incidence of PSD was analyzed. Our results showed that mean seed infection by *D*. *longicolla* was more severe after delayed harvest compared to prompt harvest at maturity. Additionally, genotypes that had low seed infection by *D*. *longicolla* when harvested on time, could show susceptibility to *D*. *longicolla* when harvest was delayed. For example, PI 594872 (MG IV) in 2007 had 2.7 and 8.0% seed infection by *D*. *longicolla* in the non-inoculated and inoculated prompt-harvest treatments, respectively. However, when harvest was delayed, the percentages of seed infection by *D*. *longicolla* for this accession were as high as 8.7% and 20.7% for the non-inoculated and inoculated treatments, respectively. Therefore, evaluating soybean genotypes under a delayed harvest regime or under conditions that favor PSD disease development is the preferred approach to identify PSD-resistant soybean lines.

Seed gemination is one of the important characteristics of seed quality. In many cases, the percent seed infection by *D*. *longicolla* is a good predictor of seed gemination. The most resistant genotypes, PI 417050 (MG II), PI 417017 (MG III), and PI 594692 (MG IV) not only had significantly (*P* ≤ 0.05) lower percent seed infected by *D*. *longicolla*, but also had higher seed germination than the other genotypes in the same maturity group. All susceptible soybean checks in the three maturity groups tested had poor germination, especially in inoculated trials with delayed harvest, which is consistent with our previous findings [9,10,29,55]. Poor seed germination of most soybean cultivars in the ESPS is typical because the current cultivar gene pool apparently lacks adequate resistance genes to PSD and heat [40]. However, identifying resistance to PSD and heat in exotic accessions, and then using those accessions to breed resistance into improved cultivars, appears to be a promising approach.

Developing and utilizing PSD-resistant cultivars is an economical and environmentally friendly strategy to protect soybean crops from disease-induced seed damage, especially when using the popular ESPS in midsouthern states. Due to an extended and committed effort, twenty-seven PSD-resistant homogeneous breeding lines with resistance to PSD and high seed quality were identified from among hundreds of breeding lines, and then tested in multi-year trials. Among these, seven soybean lines, 11043-225-72, 11043-224-91, 11030-541-28, 10061-236-11, 10076-121-11, DS65-1, and DS31-243, were transferred to public soybean breeding programs for developing improved cultivars and germplasm lines. DS31-243 (PI 700941) was publicly released by the USDA in 2022. It is expected that this research will lead to future releases of improved germplasm lines and cultivars with PSD resistance and high seed quality through the use of one or more of the above seven lines, which are all available from the ARS authors to public and commercial researchers through material transfer agreements. In terms of recommendations, DS65-1 and DS31-243 represent the best of these lines derived solely from PI 587982A, whereas 11043-225-72 is the best line derived from PI 417050 and PI 587982A, and 11030-541-28 is the best line derived from PI 424324B and PI 587982A.

*D*. *longicolla* has been identified as the main causal agent of PSD. However, although it was the dominant species in isolates from soybean in multiple studies [11–13], several different fungi in the *Diaporthe-Phomopsis* complex have also been found to be associated with PSD in soybean [11,71,72]. Evaluating soybean breeding lines with a panel of different fungi from the *Diaporthe/Phomopsis* complex would aid in the management of soybean seed decay, breeding for resistance, and be a benefit to soybean producers and the industry at large.
